# An integrated analysis of air pollution and meteorological conditions in Jakarta

**DOI:** 10.1038/s41598-023-32817-9

**Published:** 2023-04-09

**Authors:** Teny Handhayani

**Affiliations:** Fakultas Teknologi Informasi, Universitas Tarumanagara, Jakarta, Indonesia

**Keywords:** Natural hazards, Computer science, Atmospheric science, Climate change, Information technology

## Abstract

Air pollution and climate change are general problems for society. This paper proposes an integrated analysis of the Air Quality Index (AQI) and meteorological conditions in Jakarta. The column-based data integration model is applied to create integrated data of the Air Quality Index and meteorological conditions. The integrated data is then used to generate a causal graph using the PC algorithm. The causal graph reveals that there exist causal relationships between pollutants and meteorological conditions, e.g, humidity, rainfall, wind speed, and duration of sunshine affect particulate matter 10 (PM$_{10}$); wind speed affects sulfur dioxide (SO$_2$); temperature affects ozone (O$_3$). The historical data records that the average wind speed is decreased and the number of unhealthy days has risen. Ozone and particulate matter are two pollutants that mainly influence poor air quality in Jakarta. The integrated data is also used to train Long Short-Term Memory (LSTM) and Gated Recurrent Unit (GRU) for forecasting. Experimental results show that LSTM using integrated data produces smaller errors for forecasting AQI and meteorological conditions.

## Introduction

Poor air quality is dangerous to civilization and the environment^1–6^. Air pollution is the largest cause of non-communicable diseases in some countries and regions, for instance, in Southeast Asia^7^. In 2020, the government of Republic Indonesia establishes that air quality is measured from the concentration of 7 parameters: particulate matter 10 (PM$_{10}$), particulate matter 2.5 (PM$_{2.5}$), sulfur dioxide (SO$_2$), nitrogen dioxide (NO$_2$), carbon monoxide (CO), ozone (O$_3$), and hydrocarbons (HC).

Air pollution might have a linkage to meteorological conditions or vice versa. Some research has been conducted to analyze the linkage of air pollutants and meteorological conditions^8–13^. A study in Taiwan reveals that temperature was associated with the incidence of CO poisoning^14^. A Bayesian Network graphical model has been used to analyze the statistical dependencies between environmental parameters, air pollution variables, and health data^15^. A study found that the maximum aerosol optical depth (AOD) in Palangka Raya, Pontianak, and Jambi happened in the dry season from July to October^16^.

The historical data of Air Quality Index (AQI) and meteorological conditions in Jakarta record some important information^17,18^. The increasing number of unhealthy days happened from 2010–2013, 2015–2018, and 2020–2021. It is understandable that in 2020, the air quality was getting better because of the limited activities during the Covid-19 outbreak. However, the number of unhealthy days raised in 2021. From 2010 to 2021, the number of unhealthy days is always higher than healthy days. This is an early warning for the society that poor air quality might worsen if it is not managed properly. The average temperature slightly increased around $0.55 ^{\circ }$C from 2013 to 2019 and the average wind speed decreased.

Correlation measures a relationship between variables. However, correlation does not imply causation^19^. It means that statistical properties alone do not determine causal structures. The causal learning methods are enable to analyze the dependence structures among variables. A study has been conducted to observe the performance of learning algorithms to learn Bayesian network structures from climate data^20^. Some studies have been done to analyze the causal effects between pollution and health. A research has revealed the causal effects between local air pollution on daily deaths^21^. Gaussian process model and information geometric causal inference criterion have been implemented to obtain the correct causal directions between air pollutants^22^. A causal inference approach named Total Events Avoided (TEA) has been used for evaluating the health impacts of an air pollution regulation^23^.

Analyzing the trend of air pollution is beneficial for the government and society to find the important factors that contribute to air quality. This research is conducted to study the causal relationships between air pollutants and meteorological conditions in Jakarta. The problem of this research is how to analyze the causal effect of air pollution and meteorological conditions in Jakarta. This paper proposes an integrated analysis of AQI and meteorological conditions using a causal learning approach. It implements the PC algorithm to generate a causal graph from a dataset. The causal graph is then used to analyze the cause and effect relationships among variables. The proposed method is useful to analyze the linkage of air pollution and meteorological conditions in Jakarta. The integrated data is also applied to train models for forecasting. This paper implements Long Short-Term Memory (LSTM) and Gated Recurrent Unit (GRU) to forecast AQI and meteorological conditions. The research contribution is an integration model to analyze the dependency relationships among variables and prediction of the future values of AQI and meteorological conditions, the case study in Jakarta.

## Methods

### Air Quality Index (AQI)

The Ministry of Environment and Forestry in the Republic of Indonesia measures the Air Quality Index (AQI) using equation (1), where *I*, $I_a$, $I_b$, $L_a$, $L_b$, and $L_x$ represent AQI score, upper limit AQI, lower limit AQI, upper limit ambient concentration, lower limit ambient concentration, and measurement results of real ambient concentration, respectively^24^. Air Quality Index (AQI) standard values are categorized as good (1–50), moderate (51–100), unhealthy (101–200), very unhealthy (201–300), and hazardous ($\ge$301).

1 $$\begin{aligned} I = \frac{I_a - I_b}{L_a - L_b}(L_x - L_b) + I_b \end{aligned}$$

### PC algorithm

A causal graph is a graphical model that represents cause and effect relationships among variables. Assume that causal information between variables can be represented by a directed acyclic graph (DAG) where the nodes represent random variables and the edges represent direct causal effects^25–28^. Each causal DAG implies a set of conditional independence relationships^25^. A simple graph $A \rightarrow B$ (i.e., *A* is a parent of *B*) represents that A is a direct cause of B. *A* is a (possibly indirect) cause of *B* only if there is a directed path from *A* to *B* (*A* is an ancestor of *B*). One of the algorithms for learning a causal graph from a dataset is the PC algorithm^26,28,29^.

The PC algorithm applies conditional independence tests to generate a causal graph from a dataset^26^. Suppose *E*, ${\hat{\rho }}$, $\alpha$ , *n*, and $\Phi (.)$ denotes the separation set, the partial correlation, the significance level, the number of samples, and the cumulative distribution function (cdf) of ${\mathcal {N}}(0,1)$, respectively. An equation (2) can be used to compute a conditional independence test for Gaussian data^30,31^. It tests a question ‘is a variable $D_{u}$ conditionally independent $D_{v}$ of given $D_{E}$?’

2 $$\begin{aligned} D_{u}\bot D_{v} \mid D_{E} \Leftrightarrow \sqrt{n - \mid E \mid - 3} \left| \frac{1}{2} \log \left( \frac{1+{\hat{\rho }}_{uv \mid E}}{1-{\hat{\rho }}_{uv \mid E}} \right) \right| \le \Phi ^{-1}(1-\alpha /2). \end{aligned}$$

The correlation coefficient of two random variables *X* and *Y* is $\rho _{XY} = \frac{\sigma _{XY}}{\sigma _X \sigma _Y}$, where $\sigma$ is standard deviation^32^. The partial correlation can be computed from correlation matrix using Eq. (3), where *A*, *B*, and *C* are random variables^33^.

3 $$\begin{aligned} {\hat{\rho }}_{AB.C} = \frac{\rho _{AB} - \rho _{AC} \rho _{CB}}{\sqrt{(1-\rho ^2_{AC})(1-\rho ^2_{CB})}} \end{aligned}$$

In general, the PC algorithm has two main steps: generating graph skeleton and orienting the edges^34^. Suppose a dataset consists of *v* variables. The first step is generating a complete undirected network consisting of *v* vertices. The conditional independence tests are run for every triplet vertices. The output of the first step is a skeleton. The information of the conditional independence test in the first step is used to orient the edges. The output of the PC algorithm is a graph represented by a Completed Partially Directed Acyclic Graph (CPDAG)^30^. The PC algorithm can be used to learn causal graphs by assuming there are no latent variables in the dataset.

### Long short-term memory (LSTM)

Long short-term memory (LSTM) is an efficient gradient-based method^35,36^. LSTM refers to a standard recurrent neural network (RNN) that has long-term memory and short-term memory. Suppose $\zeta$, $X_t$, ${\tilde{S}}_t$, $S_{t-1}$, $S_t$, $\circ$, $O_t$ denote the sigmoid function, the preprocessed data, the new state of memory cell, the previous state of the memory cell, the final state of memory cell, Hadamard product, and the final output of the memory unit, respectively. Let $i_t$, $f_t$, $o_t$ be the output of different gates and $W^{(i)}$, $W^{(f)}$, $W^{(o)}$, $W^{(c)}$, $U^{(i)}$, $U^{(f)}$, $U^{(o)}$, $U^{(c)}$ be coefficient matrices. The mathematical models related to the LSTM memory unit are defined by Eqs. (4–9)^37^. LSTM networks work well for making predictions based on time series data^38–42^.

4 $$\begin{aligned} i_t= & {} \zeta \left( W^{(i)} X_t + U^{(i)} S_{t-1}\right) \end{aligned}$$

5 $$\begin{aligned} f_t= & {} \zeta \left( W^{(f)} X_t + U^{(f)} S_{t-1}\right) \end{aligned}$$

6 $$\begin{aligned} o_t= & {} \zeta \left( W^{(o)} X_t + U^{(o)} S_{t-1} \right) \end{aligned}$$

7 $$\begin{aligned} {\tilde{S}}_t= & {} \tanh \left( W^{(c)} X_t + U^{(c)} S_{t-1}\right) \end{aligned}$$

8 $$\begin{aligned} S_t= & {} f \circ S_{t-1} + i_t \circ {\tilde{S}}_t \end{aligned}$$

9 $$\begin{aligned} O_t= & {} o_t \circ \tanh (S_t) \end{aligned}$$

### Gated recurrent unit (GRU)

Gated recurrent unit (GRU) is recurrent neural networks (RNN) using gating mechanism^43,44^. Let $W_z$, $W_r$, *W*, $U_z$, $U_r$, *U*, $b_z$, $b_r$, and *b* be model parameters. Suppose $\odot$ represents element-wise multiplication. For each *j*-th hidden unit, GRU has a reset gate $r^j_t$ and an update gate $z^j_t$ to control the hidden state $h^j_t$ at each time *t* which are computed using Eqs. (10–13). GRU has been successfully implemented for forecasting the time series datasets^45–47^.

10 $$\begin{aligned} r_t= & {} \zeta \left( W_r x_t + U_r h_{t-1} + b_r \right) \end{aligned}$$

11 $$\begin{aligned} z_t= & {} \zeta \left( W_z x_t + U_z h_{t-1} + b_z \right) \end{aligned}$$

12 $$\begin{aligned} {\tilde{h}}_t= & {} \tanh \left( W x_t + U(r_t \odot h_{t-1}) + b \right) \end{aligned}$$

13 $$\begin{aligned} h_t= & {} (1-z_t) \odot h_{t-1} + z_t \odot {\tilde{h}}_t \end{aligned}$$

### Evaluation metric

The evaluation metric for forecasting are mean absolute error (MAE), mean square error (MSE), and root mean square error (RMSE). MAE reflects the actual situation of the prediction error and RMSE evaluates the degree of change and accuracy of the data. Let $y'$, *y*, and *n* be the predicted value, true value, and the number of samples. Equations (14–16) are used to compute MAE, MSE, and RMSE, respectively^47,48^.

14 $$\begin{aligned} MAE_{y', y}= & {} \frac{1}{n} \sum _{i = 1}^{n}|y'_i - y_i | \end{aligned}$$

15 $$\begin{aligned} MSE_{y', y}= & {} \frac{1}{n} \sum _{i = 1}^{n}(y'_i - y_i)^2 \end{aligned}$$

16 $$\begin{aligned} RMSE_{y', y}= & {} \sqrt{\frac{1}{n} \sum _{i = 1}^{n}(y'_i - y_i)^2} \end{aligned}$$

### Dataset

This paper use AQI and meteorological conditions in Jakarta from public datasets. The AQI data owned by DKI Jakarta Provincial Government can be accessed at https://data.jakarta.go.id/organization/badan-pengelolaan-lingkungan-hidup-daerah^18^. The meteorological conditions dataset is obtained from an open dataset belonging to Indonesian Agency for Meteorological, Climatological and Geophysics (Badan Meteorologi, Klimatologi, dan Geofisika or simply BMKG) that is available at http://dataonline.bmkg.go.id/home^49^.

The air quality dataset is daily records Air Quality Index (AQI) of PM$_{10}$, PM$_{2.5}$, SO$_2$, CO, O$_3$, and NO$_2$ from 2010 to 2021. PM$_{2.5}$ is only available from 2021. The meteorological conditions is a daily record of average temperature ($^\circ$C), average relative humidity (RH) (%), average rainfall (mm), average duration of sunshine (hours), and average wind speed (m/s) from 2010 to 2021.

### The proposed method

**Figure 1:**
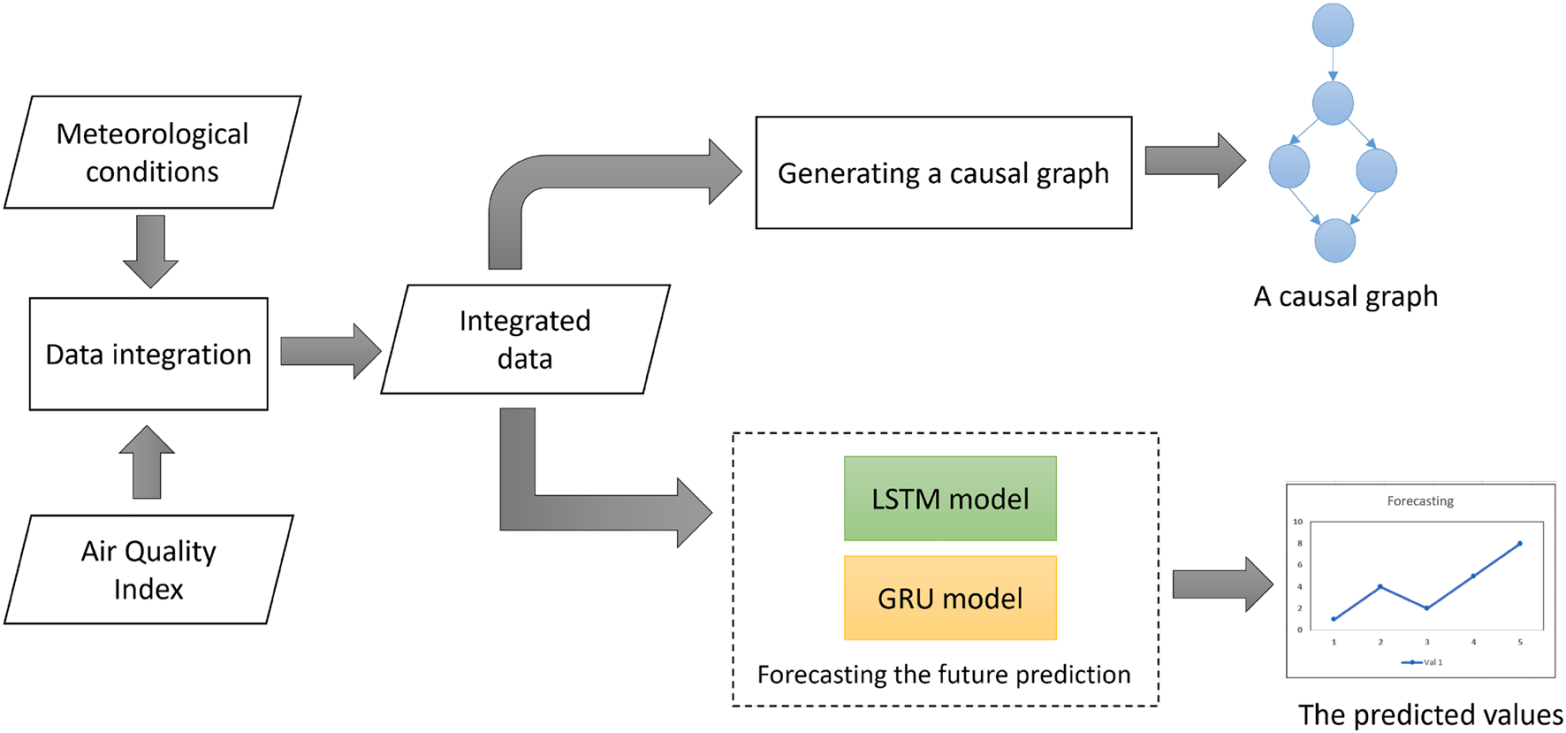
The proposed method of an integration analysis of air pollution and meteorological conditions.

This paper proposes an integrated analysis of air pollution data and meteorological condition to analyze the air quality in Jakarta. The proposed method is illustrated in Fig. 1. The stages of the proposed method are data integration, causal graph generation, and forecasting. The integration process of meteorological data and AQI data use column-based integration. The datasets are time series data with numerical values. The idea of data integration has been used to learn simultaneously from multiple data sources^50,51^. The integration data requires not only the same date for each sample but also the same number of samples from all resources. In this paper, the integrated data is a single table containing variables from meteorological data and AQI data. This data is then used as input for generating a causal graph and forecasting. A causal graph is generated using the PC algorithm. LSTM and GRU are implemented for forecasting.

This paper uses the PC algorithm from *bnlearn* in the R package^52,53^. A causal graph is generated in R Studio. It also implements LSTM and GRU from TensorFlow Keras. The forecasting is run in Jupyter Notebook for Python.

LSTM and GRU are implemented to forecast the prediction of AQI and meteorological conditions. The LSTM and GRU models consist of stacked layers with 128 and 64 units, dropout layer and dense layer. LSTM and GRU are run for 50 epochs and they implement the Softmax activation function. This paper uses multivariate forecasting. The experiments use integrated and not integrated data. The letter *i* and *p* indicate that the algorithm is implemented for forecasting using integrated data and not integrated data, respectively. A not-integrated data refers to AQI data or a meteorological conditions dataset. An integrated dataset is a dataset containing AQI and meteorological conditions obtained from data integration process. This paper runs multivariate forecasting in 3 different scenarios:

- Experiment 1 using training set from 2010 to 2018 and testing set from 2019.
- Experiment 2 using training set from 2010 to 2019 and testing set from 2020.
- Experiment 3 using training set from 2010 to 2020 and testing set from 2021.

## Results and discussion

The datasets are containing less than 5% missing values. The missing values are filled up using an average value of the observed variables from 7 days before the observed date. After preprocessing phase, it implements column-based integration to create a single formed data from AQI and meteorological condition datasets. The integrated data is used to generate a causal graph and to train models for forecasting.

### Causality analysis

**Figure 2:**
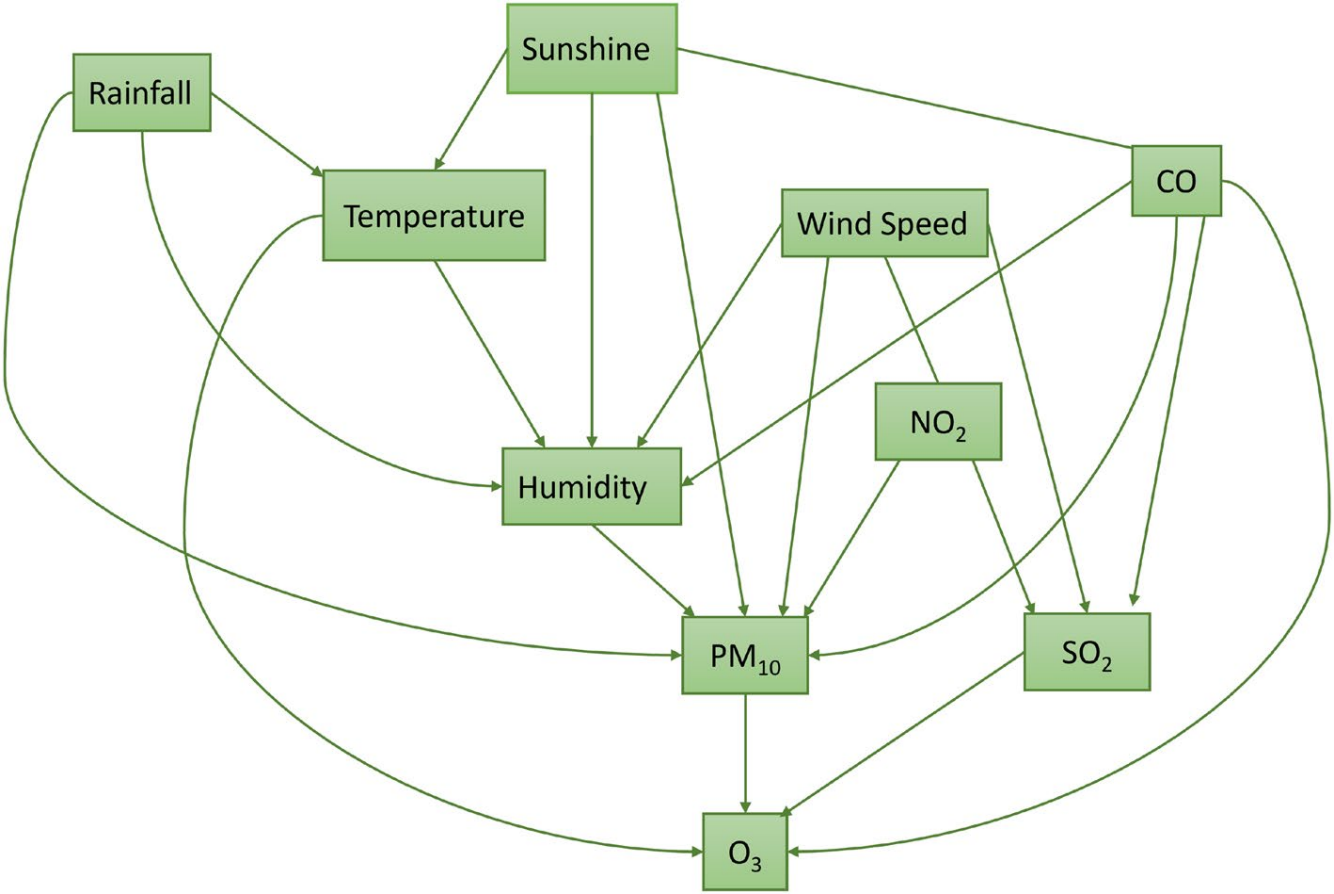
A causal graph is generated from AQI and meteorological conditions.

This paper examines the dependence relationships between air pollutants represented by AQI and meteorological conditions. A graph is generated from an integrated data of AQI and meteorological conditions from 2010 to 2021. The dataset consists of 4383 samples and 10 variables (temperature, humidity, rainfall, sunshine, wind speed, PM$_{10}$, SO$_2$, CO, O$_3$, and NO$_2$). PM$_{2.5}$ is not included to the experiments due to the samples are only available from 2021. Figure 2 shows a causal graph generated using the PC algorithm at significance level of $\alpha = 0.05$. The graph finds some information that will be explained as follows.

- Humidity, rainfall, and duration of sunshine are causal parameters for PM$_{10}$. Those findings are corresponding to some previous studies. Humidity influences PM’s natural deposition process; moisture particles adhere to PM and accumulate atmospheric PM concentration^9^. The increasing humidity reduces PM$_{10}$ concentrations in the atmosphere because moisture particles grow in size to a point where ‘dry deposition’ happens. PM$_{10}$ continually reduced with humidity rising^10^. The precipitation has a certain wet scavenging effect on PM$_{2.5}$ and PM$_{10}$^11^. Precipitation scavenging refers to the cleaning of gases and particles by cloud and precipitation elements. A study of ambient air quality in Jakarta found that the concentration of suspended particulate matter is decreased in the wet season (October–March) and increased in the dry season (April–September) because rainfall removes the pollutant in the atmosphere^54^.
- CO has a dependent relationship to humidity. The previous study shows that higher humidity has a negative effect on the adsorption of carbon monoxide^55^.
- Wind speed has dependence relationships to SO$_2$, NO$_2$, and PM$_{10}$.
- Temperature has a causal relationship to O$_3$. The chemical reactions in the formation or destruction of O$_3$ are influenced by temperature, solar radiation, and wind speed^56^. A study found that diurnal temperature range, precipitation, and wind speed had the largest impact on SO$_2$ in Shandong, China^57^.
- CO causes O$_3$ and it is similar to a study in Kota Bharu, Malaysia that discovers CO as a causal parameter for O$_3$^58^.
- PM$_{10}$, SO$_2$, and CO affect O$_3$. O$_3$ is an air pollutant that is formed in the atmosphere from a combination of nitrogen oxides, volatile organic compounds, CO, and methane in the presence of sunlight^59^.
- NO$_2$ affects PM$_{10}$ and SO$_2$.
- Sunshine affects PM$_{10}$.

The causal graph in Fig. 2 explains the connection of certain parameters from meteorological conditions to air pollution. Those relationships are not revealed when the analysis is done separately.

### Correlation analysis

This paper highlights correlation coefficient ($\rho$) with the values $\rho \ge \pm 0.2$. The correlation coefficient between variables except for PM$_{2.5}$ is computed from samples of 2010–2021. The correlation coefficient involving PM$_{2.5}$ is obtained from the dataset of 2021. Table 1 shows correlation coefficient between two variables computed using Pearson correlation. The longer the sunshine duration makes the higher temperature, lower humidity, and lower rainfall. The temperature and duration of sunshine have a positive correlation to PM$_{10}$ and PM$_{2.5}$. The more concentration of PM the higher temperature will be. Humidity has a negative correlation to PM$_{10}$ and PM$_{2.5}$. This is one of the possible ways to decrease PM concentration by increasing humidity. Higher rainfall increases humidity. Weather modification to create artificial rain is useful to decrease PM concentration. Humidity and CO have a positive correlation. Meanwhile, wind speed and SO$_2$ have a negative correlation.

**Figure 3:**
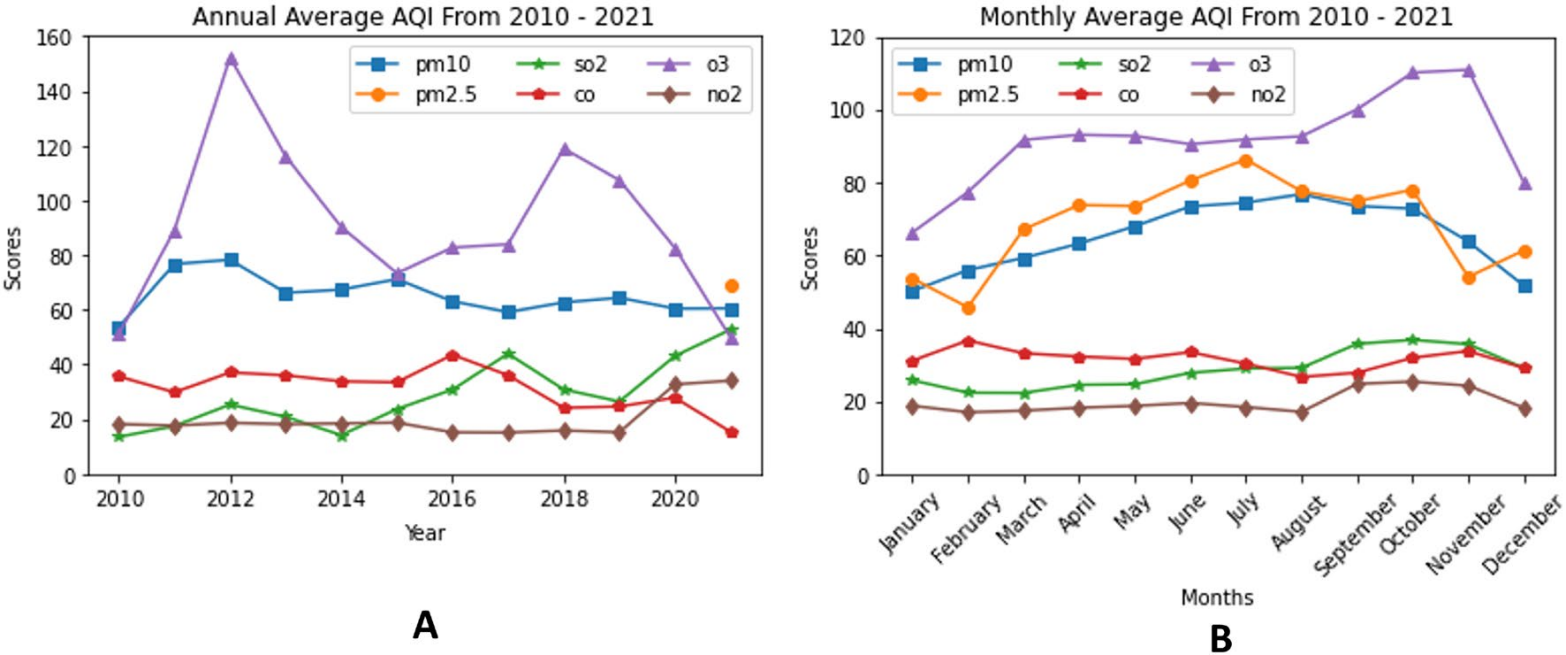
The (**A**) annual and (**B**) monthly average AQI in Jakarta from 2010 to 2021.

The annual average AQI from 2010 to 2021 is illustrated in Fig. 3A. The highest exposure to O$_3$ happened in 2012. Figure 3B shows the monthly average AQI in Jakarta from 2010 - 2021. The top 3 air pollutant are O$_3$, PM$_{2.5}$ and PM$_{10}$. AQI score of O$_3$ is always higher than 50 and it reaches over 100 in October to November which is categorized as an unhealthy condition.

**Table 1:** Correlation coefficient.

	Temperature	Humidity	Rainfall	Sunshine	Wind	PM$_{10}$	SO$_2$	CO	O$_3$	NO$_2$	PM$_{2.5}$
Temperature	1	− 0.69	− 0.38	0.47	− 0.02	0.31	0.12	− 0.12	0.17	0.03	0.41
Humidity	− 0.69	1	0.40	− 0.49	− 0.06	− 0.35	− 0.12	0.25	− 0.16	− 0.02	− 0.34
Rainfall	− 0.38	0.40	1	− 0.21	− 0.03	− 0.20	− 0.07	0.07	− 0.07	− 0.02	− 0.36
Sunshine	0.47	− 0.49	− 0.21	1	− 0.02	0.27	0.09	− 0.18	0.13	0.02	0.19
Wind	− 0.02	− 0.06	− 0.03	− 0.02	1	− 0.05	− 0.32	0.02	0.01	− 0.10	− 0.32
PM$_{10}$	0.31	− 0.35	− 0.20	0.27	− 0.05	1	0.05	0.19	0.35	0.12	0.73
SO$_2$	0.12	− 0.12	− 0.07	0.09	− 0.32	0.05	1	− 0.12	− 0.06	0.60	0.33
CO	− 0.12	0.25	0.07	− 0.18	0.02	0.19	− 0.12	1	0.15	0.05	0.19
O$_3$	0.17	− 0.16	− 0.07	0.13	0.01	0.35	− 0.06	0.15	1	0.00	0.32
NO$_2$	0.03	− 0.02	− 0.02	0.02	− 0.1	0.12	0.60	0.05	0.00	1	0.14
PM$_{2.5}$	0.41	− 0.34	− 0.36	0.17	− 0.32	0.73	0.33	0.19	0.33	0.14	1

Sunrise and sunset in Jakarta are not significantly different every day throughout the year because it lies on a latitude of − 6$^\circ$12$^\prime$ 52.63$^\prime$ $^\prime$ S and a longitude of 106$^\circ$50$^\prime$ 42.47$^\prime$ $^\prime$ E. The length of daylight remains the same every day, so the duration of sunshine is mostly affected by clouds. In the last 10 years, the low average rainfall happens from May to September, and the lowest is around 1.8 mm in August. Meanwhile, the longest average sunshine duration occurs in August, September, and October at 5.7, 6.4, and 5.3 hours, respectively. The month of June to October has a high average level of PM$_{10}$ over 73 and the highest is 76.79 in August. The lowest average of PM$_{10}$ is 50.24 in January. This finding is closed to the previous study^54^ which is states that the highest concentration of PM$_{10}$ occurs in September 2015 and the lowest one is in February 2017. In 2021, the two highest average AQI for PM$_{2.5}$ are 80.56 in June and 86.32 in July. In May and October, the average temperature is around 29.1 $^\circ$C which is higher than the overall average temperature of 28.49 $^\circ$C. The average humidity during July–October is around 70–74%. Since 2015, the average wind speed decreases around 1 m/s than that in 2010. In 2021, the correlation between wind speed and PM$_{2.5}$ $\rho$(wind speed and PM$_{2.5}$) is − 0.32. The decrement in wind speed contributes to increasing PM$_{2.5}$. Wind speed and SO$_2$ have a negative correlation, so decreasing wind speed rises SO$_2$. A positive correlation is obtained between SO$_2$ and NO$_2$ as 0.6, indicating that the concentration of those pollutants rises together. O$_3$ has a positive correlation to PM$_{10}$ and PM$_{2.5}$.

### The historical data and forecasting models

A record of the number of unhealthy (U) and very unhealthy days (VU) in the year 2010–2021 is presented in Table 2. The historical data shows that O$_3$ is the pollutant that mostly causes unhealthy and very unhealthy days. There are 108 days where on the same day two pollutants have AQI scores over 100 but only 22 days were labeled as very unhealthy because they only pay attention to a pollutant that has the highest AQI scores on that days. In 2020, on three consecutive days, the three pollutants together (SO$_2$, O$_3$, and NO$_2$) have AQI scores of more than 100 and those are categorized as unhealthy. It needs further study for a case when more than two pollutants have AQI scores over 100 in a day. It is possible to be more hazardous when the concentration of multiple pollutants reaches the unhealthy limit at the same time, so the categories of air pollution levels need to be evaluated.

**Table 2:** Pollutants that affect unhealthy (U) and very unhealthy (VU) days in Jakarta.

Year	${}_{U}$O$_3$	${}_{U}$CO	${}_{U}$PM$_{2.5}$	${}_{U}$PM$_{10}$	${}_{U}$SO$_2$	${}_{U}$NO$_2$	${}_{VU}$O$_3$
2010	18	0	0	0	0	0	4
2011	105	0	0	41	0	0	5
2012	123	0	0	25	0	0	116
2013	177	0	0	4	0	0	27
2014	83	1	0	6	0	0	8
2015	43	0	0	21	0	0	0
2016	48	0	0	0	0	0	1
2017	105	0	0	5	0	0	0
2020	57	25	0	1	8	10	3
2021	2	0	136	2	1	1	0

**Figure 4:**
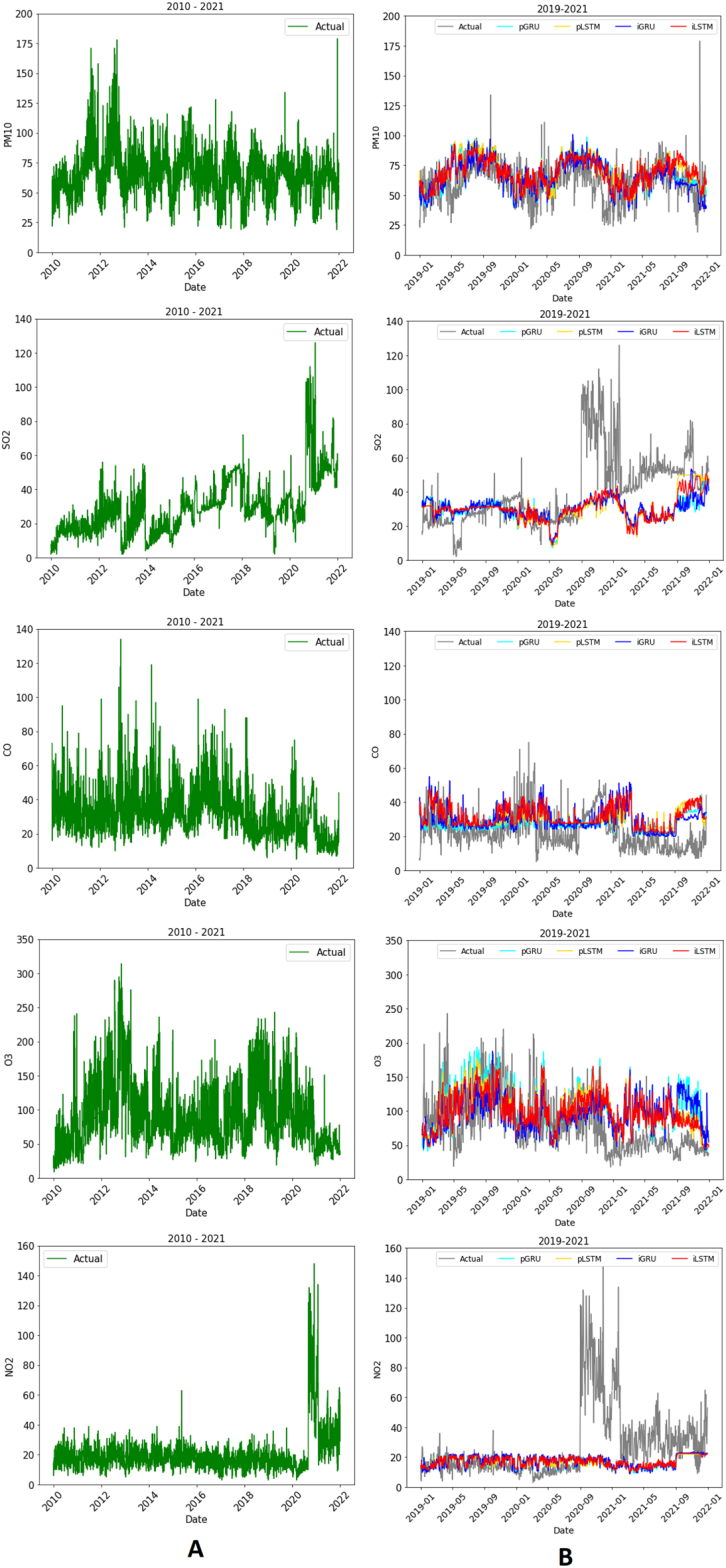
The actual data (**A**) and forecasting (**B**) of AQI in Jakarta.

**Figure 5:**
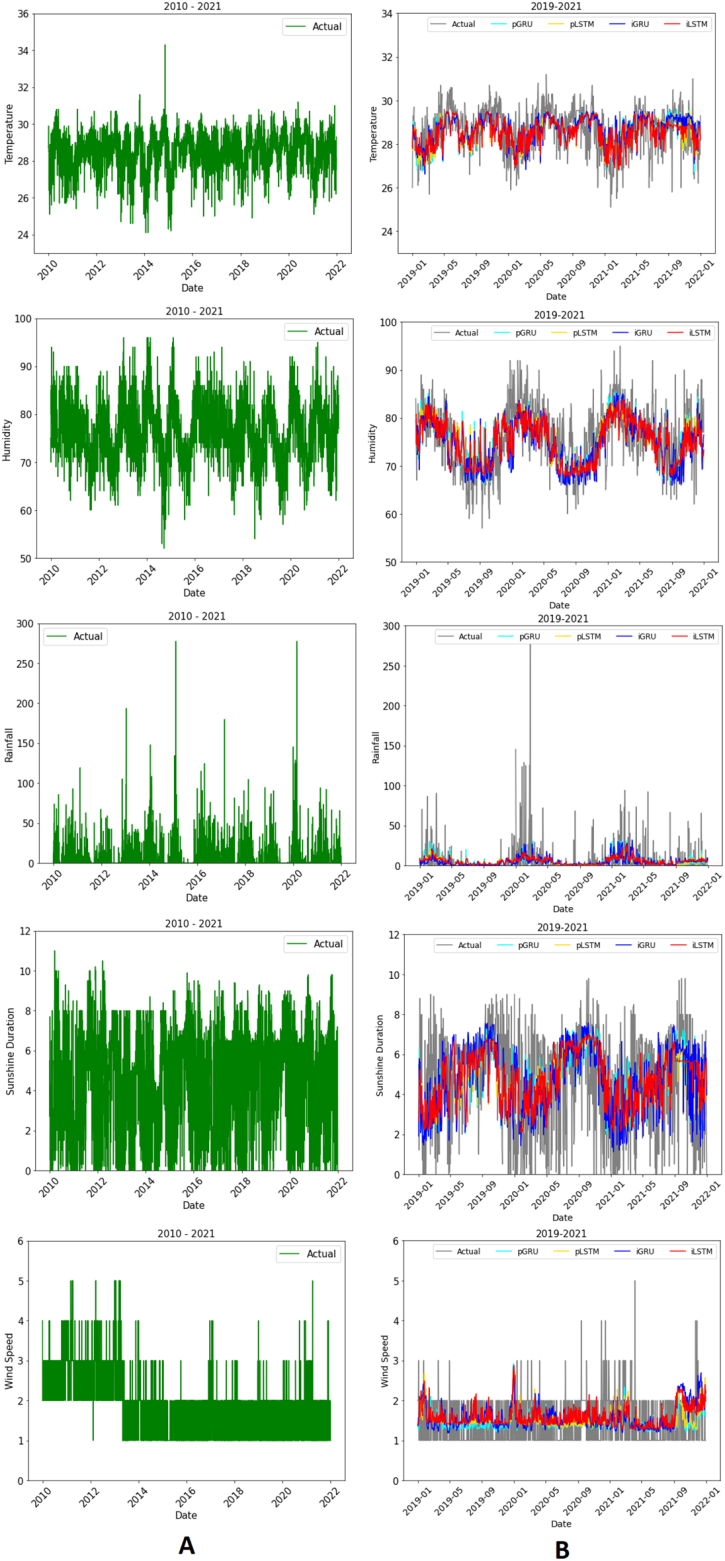
The actual data (**A**) and forecasting (**B**) of meteorological condition in Jakarta.

The previous studies reveal various effects of the pollutants. The ambient temperature increased acute cardiovascular-respiratory mortality effects of PM$_{2.5}$^60^. Exposure to PM$_{10}$, NO$_2$, and O$_3$ generates a relative risk to human health^61^. The effect of humans inhaling O$_3$ possibly leads to acute lung function changes and inflammation^62^. PM$_{2.5}$ may contribute to the development of diabetes mellitus, increase cardiopulmonary morbidity and mortality, and cause adverse birth outcomes^63^. Epidemiological evidence shows that PM$_{2.5}$ damage the human respiratory system^64^. The accumulating of exposure to low concentrations of carbon monoxide can affect a number of organ systems^65^.

**Figure 6:**
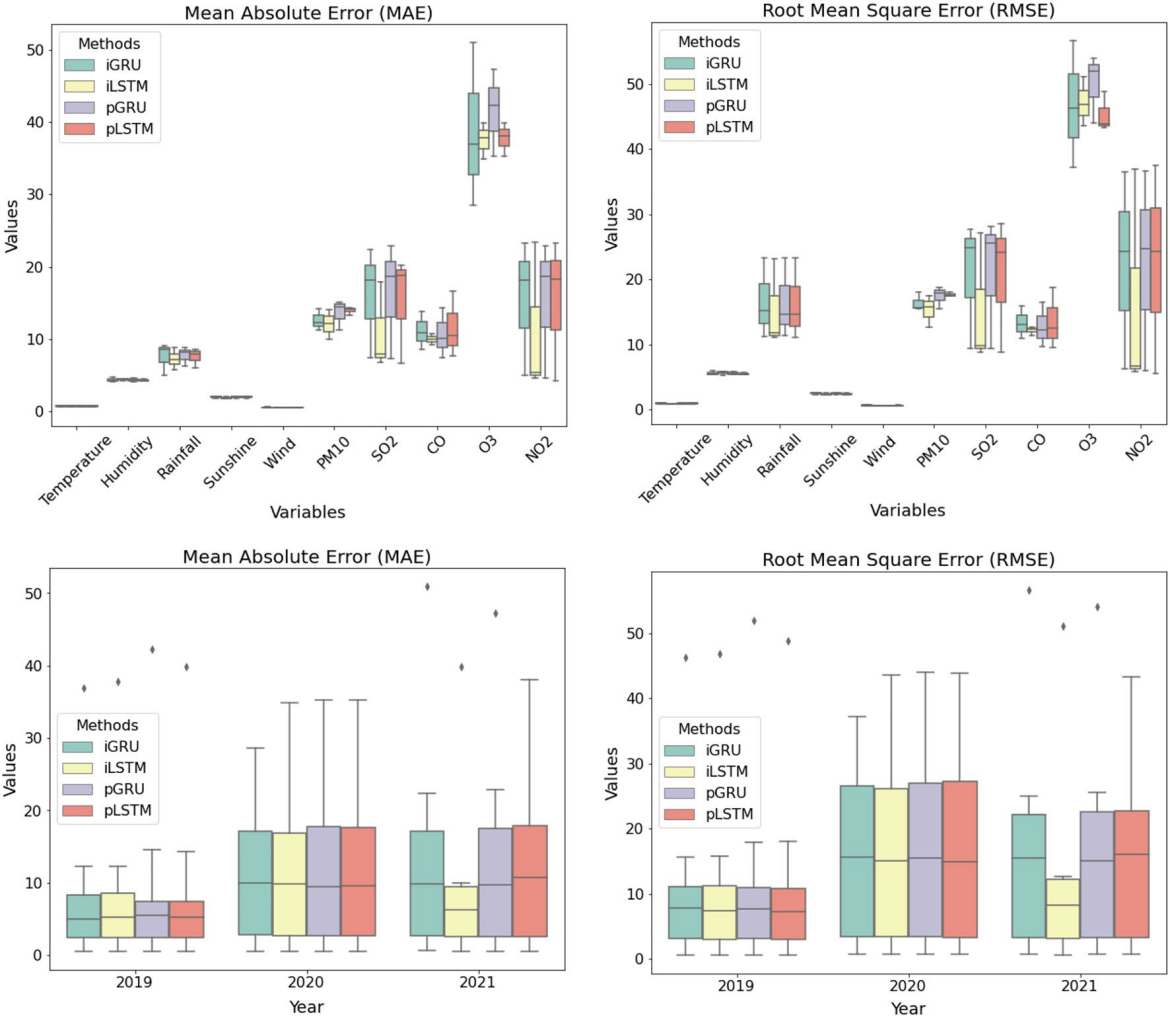
A comparison performance using integrated data (*i*) and not integrated data (*p*).

The actual data and forecasting of AQI from 2010 to 2021 are described in Fig. 4 A and B, respectively. The performance of LSTM and GRU are evaluated using MAE and RMSE. According to the experimental results, LSTM using integrated data produces the smaller error. In general, LSTM and GRU show a good performance in forecasting PM$_{10}$, CO, and O$_3$.

The actual data and forecasting meteorological conditions are described in Fig. 5A and B, respectively. LSTM and GRU work well to forecast temperature, humidity, sunshine duration and wind speed. However, they are less accurate to predict rainfall.

The two highest AQI of PM$_{10}$ were in 2011 and 2013 when the averages were 76.59 and 78.21. The AQI of SO$_2$ was consistently rising around 3 times higher than in 2010. The AQI of CO increased from 2010 to 2017, but it decreased from 2020 to 2021. The AQI of O$_3$ was also rising and the highest was in 2012–2013. Figure 6 shows the values of MAE and RMSE for forecasting results. LSTM using integrated data produces smaller errors. In general, the forecasting results of AQI data from 2020 to 2021 have higher errors than that from 2019. It is suspected that major restrictions in some activities during the Covid-19 outbreak influence that condition, for instance, the national or local lockdown reduces the use of motor vehicles which decreases the CO level. There was a huge increase in SO$_2$ and NO$_2$ from September 2020–January 2021 but the reason is unknown. It needs further study for investigation. Comparing to the other study which is forecasting the observed variables using not integration data^66^, the forecasting using integration data produces slightly lower MAE and RMSE.

The findings in this paper are expected to enrich the knowledge of the linkage between air pollution and climate change. This contribution is beneficial to determining the proper handling of air pollution and climate change problems.

## Conclusion

In conclusion, the integration analysis successfully discovers the linkage between air pollution and meteorological conditions in Jakarta. The integrated data is used to generate a causal graph and to train models for forecasting. A causal graph shows that there exist dependence relationships between AQI and meteorological conditions. This information is beneficial for handling air pollution and climate change. LSTM and GRU work well as models for forecasting PM$_{10}$, CO, O$_3$, temperature, humidity, sunshine duration, and wind speed. However, those models show less accurate to predict SO$_2$, NO$_2$, and rainfall. LSTM using integrated data produces a smaller error. The forecasting results of air pollution before the Covid-19 outbreak are more accurate. The Covid-19 outbreak influences human activities that probably affect air quality, e.g, decreasing CO, and increasing NO$_2$ and SO$_2$. The future work is implementing machine learning approach for an integrated analysis to find the connection of population growth, industries, human activities and air pollution to the climate change in Indonesia.

## Data Availability

The datasets are available from the corresponding author by request for strong reasons.
